# 3D Photon-To-Digital Converter for Radiation Instrumentation: Motivation and Future Works

**DOI:** 10.3390/s21020598

**Published:** 2021-01-16

**Authors:** Jean-François Pratte, Frédéric Nolet, Samuel Parent, Frédéric Vachon, Nicolas Roy, Tommy Rossignol, Keven Deslandes, Henri Dautet, Réjean Fontaine, Serge A. Charlebois

**Affiliations:** Interdisciplinary Institute for Technological Innovation and Department of Electrical and Computer Engineering, Université de Sherbrooke, Sherbrooke, QC J1K 2R1, Canada; Samuel.Parent@USherbrooke.ca (S.P.); Frederic.F.Vachon@usherbrooke.ca (F.V.); Nicolas.Roy6@Usherbrooke.ca (N.R.); Tommy.Rossignol@USherbrooke.ca (T.R.); Keven.Deslandes@USherbrooke.ca (K.D.); Henri.Dautet@USherbrooke.ca (H.D.); Rejean.Fontaine@usherbrooke.ca (R.F.); Serge.Charlebois@USherbrooke.ca (S.A.C.)

**Keywords:** single-photon avalanche diode, SPAD array, SiPM, silicon photomultiplier, digital SiPM, 3D photon-to-digital converter, 3D heterogeneous integration, time-of-flight, positron emission tomography, liquid argon, liquid xenon

## Abstract

Analog and digital SiPMs have revolutionized the field of radiation instrumentation by replacing both avalanche photodiodes and photomultiplier tubes in many applications. However, multiple applications require greater performance than the current SiPMs are capable of, for example timing resolution for time-of-flight positron emission tomography and time-of-flight computed tomography, and mitigation of the large output capacitance of SiPM array for large-scale time projection chambers for liquid argon and liquid xenon experiments. In this contribution, the case will be made that 3D photon-to-digital converters, also known as 3D digital SiPMs, have a potentially superior performance over analog and 2D digital SiPMs. A review of 3D photon-to-digital converters is presented along with various applications where they can make a difference, such as time-of-flight medical imaging systems and low-background experiments in noble liquids. Finally, a review of the key design choices that must be made to obtain an optimized 3D photon-to-digital converter for radiation instrumentation, more specifically the single-photon avalanche diode array, the CMOS technology, the quenching circuit, the time-to-digital converter, the digital signal processing and the system level integration, are discussed in detail.

## 1. Introduction

A recently published special issue [1] presented and discussed the various aspects and key parameters of silicon photomultipliers (SiPMs), or more precisely of analog SiPMs. This type of photodetector, introduced by Saveliev and Golovin [2], revolutionized the field of radiation instrumentation by gradually replacing both avalanche photodiodes and photomultiplier tubes in many systems. Analog SiPMs nevertheless have limitations which begs the question: what is the next photodetector technology that will replace analog SiPMs and be a game changer in radiation instrumentation experiments? This paper focuses on 3D photon-to-digital converters (PDCs), also known as 3D digital SiPMs, and positions this disruptive technology in the field of radiation instrumentation.

The focus on radiation instrumentation in this paper sets the wavelength range of interest to ~500 nm and below. This covers the range of scintillation photons currently used for positron emission tomography (PET) imaging (LaBr_3_: 360 nm and 380 nm; LSO 420 nm; BGO 505 nm) [3,4], Cherenkov radiation [5,6] for future PET with time-of-flight capability [7] as well as for Cherenkov telescope arrays (CTAs) [8] where the emission spectrum follows a $1/\lambda^{2}$ dependence [5,9], large-scale liquid xenon (LXe) detectors ( 175 nm) [10,11] and liquid argon (LAr) detectors (128 nm) without wavelength shifter (~420 nm with wavelength shifter) [12,13,14,15], only to name a few [16,17]. Therefore, the discussion does not relate to wavelengths in the infrared that are nevertheless a very hot topic for light detection and ranging (LiDAR) using CMOS single-photon avalanche diode (SPAD) arrays for autonomous vehicles.

The paper first reviews the basic structure of SPAD arrays and discusses how to take advantage of them from a digital point of view in Section 2 and Section 3. This will lead into Section 4 with a discussion on taking advantage of 3D integration of the More than Moore trend by integrating heterogeneous microelectronics and optoelectronics technologies vertically, where the SPAD array and the front-end electronics are stacked on distinct physical tiers. Even though the 3D PDC is an emerging technology in the field of radiation instrumentation, the first implementation of such devices dates to the beginning of the 2000s from the MIT Lincoln Laboratory [18,19,20], and hence in Section 5, a review of existing 3D PDCs will be presented. Then, in Section 6, the R&D of the main building blocks of the 3D PDC will be discussed, including system level considerations.

## 2. SPAD, Analog SiPM and PDC

The SPAD is the basic unit cell of all SiPM/PDC technologies [21,22]. It consists of a photodiode biased above breakdown in a metastable state awaiting to be triggered by a photoelectron or a thermally excited carrier to switch to high current. Once triggered, a resistor or a CMOS circuit quenches the current streaming through the photodiode, which reduces the photodiode’s bias below the breakdown voltage. A SPAD is a non-linear photodetector by nature because its response is the same whether one or many photons have triggered it [23,24]. The SPAD’s response is Boolean: it is either waiting for a carrier in the metastable state or triggered.

**Figure 1:**
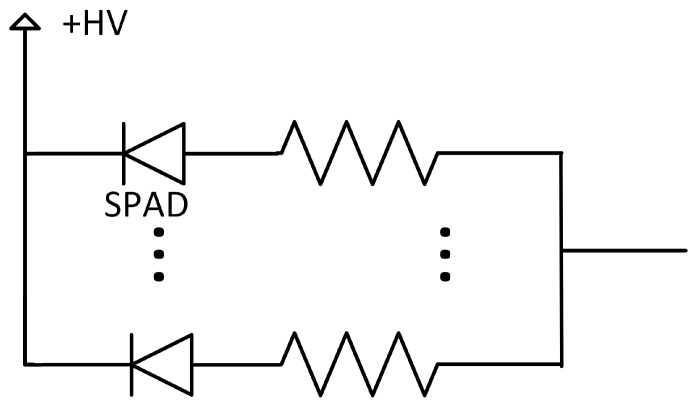
Schematic of an analog SiPM.

**Figure 2:**
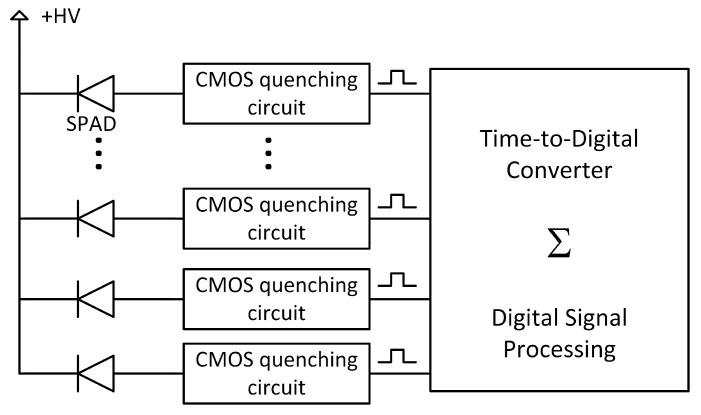
Schematic of a PDC. A CMOS microelectronic circuit is used to quench 1-to-1 a given SPAD.

From this non-linear Boolean response, an analog SiPM gives out a linear response by performing an analog sum of the current in each SPAD-resistor pair (Figure 1). The response is linear only for low photon flux. In situations where there are more photons than the number of available SPADs as a function of time (i.e., not recharging), energy measurement needs to be corrected. In a PDC, the number of triggered SPADs can be obtained in many ways, with the most common being a digital counter summing the digital output of the CMOS quenching circuits as shown in Figure 2.

### Historical Review of SPAD-Based Photodetector

From 1950 to 1960, most SPAD research was on studying the micro-plasma that is created in a p-n junction and which is the foundation of the avalanche breakdown process [25,26,27,28]. In the following years, the term avalanche photodiode emerged [29] and, at the beginning of the 70s, the first avalanche photodiodes were commercially available from RCA [30]. In parallel, Cova and his team started working on SPADs and invented the quenching circuit (QC) to build a single-photon detector [31,32]. The first SPAD-based systems were implemented using discrete electronic components to form a single-channel SPAD detector [33]. Their performance in terms of noise, speed and timing measurement accuracy were limited by the large capacitance of a long interconnection (∼cm) between the discrete SPAD and the readout quenching circuit [34]. During the 80s and the 90s, the field progressed significantly in understanding the SPAD nuisance parameters and overall characteristics. In 1998, the MIT Lincoln Lab was the first to build a 4×4 SPAD array that was piggybacked on a 16-channel readout ASIC for a LiDAR application [18,20]. Still at that point, each SPAD was wirebonded to its readout channel. To lower the capacitance between the SPAD and its readout circuit, the next major step came when single-photon detectors took the avenue of microelectronics integration, with the goal to integrate both the SPAD and readout quenching circuit into a single commercial microelectronics process [35,36,37,38,39,40]. These innovations led into the early 2000s to the first multi-pixel SPAD arrays, grouped in this review under 2D PDCs [41,42]. 2D PDCs have progressed tremendously since then and the literature is abundant on the topic (see references in [43]). It is interesting and counterintuitive to note that the first analog SiPMs were presented by Saveliev and Golovin in 2000 [2] while a lot of work was already ongoing on the digital approach.

## 3. Analog versus Digital SPAD Array

In this section, analog SiPMs and PDCs are compared with highlights on their major differences.

### 3.1. Paradigm Shift or Back to the Start?

To read out an analog SiPM, one must use a charge sensitive preamplifier, a transimpedance amplifier or a current amplifier [44]. The signal is then shaped to obtain single-photon resolution with proper signal-to-noise ratio. Finally, the signal is digitized by an analog-to-digital converter. However, one must realize that the Boolean information about the state of each SPAD (triggered or not) was already available at the SPAD level. Therefore, analog SiPMs convert Boolean information into an analog signal that is then digitized again at the cost of power consumption and complex analog readout. On the contrary, in a PDC, each SPAD is read out individually by its own quenching circuit, acting like a leading-edge discriminator and outputting a binary signal corresponding to the SPAD state. At that point, proper digital signal processing can be implemented to meet the application goal.

### 3.2. Reading Out Each SPAD Individually

Reading out each SPAD individually in a PDC has significant advantages. Within a SPAD array, the breakdown voltage varies from SPAD to SPAD due to fabrication process deviations, temperature variation and IR drop that is a function of the position [45]. When performing single-photon counting with analog SiPMs, these fluctuations blur the spectrum at high photon counts. With a PDC, each triggered SPAD raises a flag processed by the digital logic [41,42,46,47,48]. Most variations in the SPAD’s response are thus masked by the digital conversion performed by the quenching circuit which therefore provides a greater SPAD-to-SPAD variation immunity. This can be exploited to achieve more accurate energy measurement, improved single-photon timing resolution (SPTR), better sensor uniformity, and lower sensitivity to temperature variation.

Both analog SiPMs and PDCs have the ability to measure single photons. In the readout chain of an analog SiPM, each stage including the analog-to-digital converter contributes to the noise of the measurement [49,50]. In a well-designed readout chain, the noise is dominated by the output capacitance of the SiPM. This can be mitigated by increasing the transconductance of the first transistor in the readout path with a large current, but at the cost of an increased power consumption [51].

With a PDC, the concept of electronic noise is irrelevant to the ability to resolve single photons. The signal-to-noise ratio of the readout chain does not scale with the capacitance of the SPAD, as opposed to analog SiPMs. A given quenching circuit sees the small capacitance of only one SPAD (~tens to hundreds of fF) and a fast-rising voltage step with an amplitude equal to the difference between the SPAD bias voltage and its breakdown voltage (known as $V_{excess}$). The quenching circuit acts as a leading-edge discriminator providing single-photon resolution.

SPADs may be damaged during fabrication, exhibit a noise count rate above specification, or get radiation damage within the experiment [52]. In an array, resolving single photons may therefore become impossible as the noise floor, i.e., the average number of triggered SPADs without excitation, increases. In extreme cases, an analog SiPM might need to be shut down with the consequence of losing photosensitive area equal to the SiPM size. Regarding radiation damage specifically, SiPMs’ radiation hardness is increased by decreasing the SPAD size at the cost of fill-factor. In the case of PDCs, noisy SPADs can be turned off or their bias voltage can be adjusted through the integrated circuit programming interface [53]. This way, the noise floor does not increase, preserving single-photon resolution, while the total dynamic range is lowered only by the number of disabled SPADs.

The photon detection efficiency (PDE) of a SPAD is proportional to its quantum efficiency at a given wavelength and to the probability of triggering a sustainable avalanche as a function of the excess voltage. With an analog SiPM, there is no control on the recharge time resulting in an effective dynamic PDE change while recharging the SPAD. With a PDC, one can swiftly recharge the SPAD with a CMOS circuit, providing greater PDE uniformity and single-photon counting.

Optical crosstalk and afterpulsing are correlated noises which pollute the signal in all types of SPAD-based detector. They are proportional to the number of charges involved in the avalanche of a SPAD [54]. Analog SiPMs have no control over the quenching and recharge of each SPAD during operation. On the contrary with PDCs, an avalanche can be detected at its beginning and be swiftly quenched, thus limiting the charges involved in the avalanche. To limit afterpulsing noise, a programmable delay can be implemented in a PDC, to keep each SPAD off over a desired period of time after it was triggered. This allows for the trapped charges to be released without triggering a new avalanche, which improves the effective PDE [55]. By programming the hold-off time and the recharge time, the correlated noise can be significantly reduced during operation, at the cost of a detection deadtime. This is a major advantage of PDCs over analog SiPMs.

### 3.3. Power Consumption Comparison

It is a complex task to compare the power consumption of systems that do not have the same approach, in particular, when the output information is in a different form. For photon counting with an analog solution, the first stage of amplification (current, transconductance or charge sensitive amplifiers), shaper, sample and hold and ADC are continuously consuming power, whether there are events or not. With PDCs based on CMOS readout circuits, the absence of events leads to very low power consumption in the quenching circuit while the digital counter logic consumption is only significant when a SPAD is triggered. The idle power consumption is set by auxiliary circuits (ex: monostable and bias circuits—depending on the integrated circuit architecture) and the transistor gate leakage current, which can be non-negligible for large-scale detectors and deep sub-micron technologies [56]. Moreover, PDCs can work synchronously with a constant clock or asynchronously where the clock is only activated when data is available. The latter option leads to a significant reduction in power consumption.

## 4. 2D versus 3D Integrated PDC

**Figure 3:**
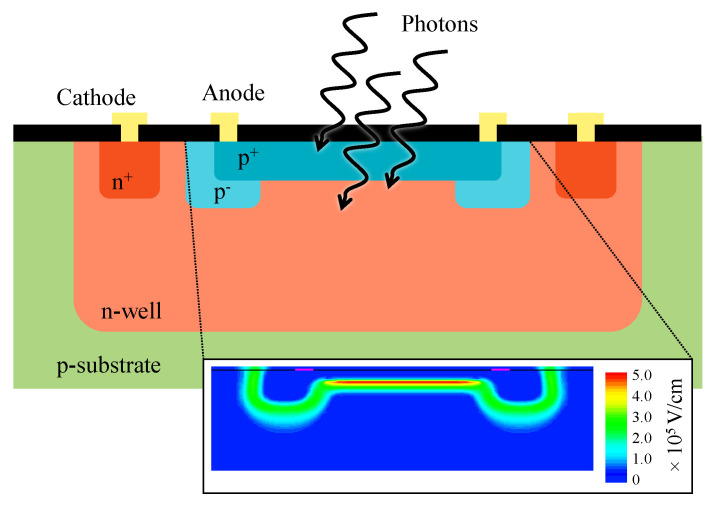
Cross-section of a CMOS SPAD. Shown here is a frontside illuminated p^+^ in n-well SPAD. The inset shows a uniform high electric field region under the whole sensitive area of the SPAD achieved by optimization of the guard-ring geometry and doping level.

A 2D PDC consists of an array of SPADs and quenching circuits integrated together in a CMOS process [41,42,46,47,57,58]. The SPAD is usually a shallow planar junction implemented using the transistor drain/source implantation in a well of the opposite type as shown in Figure 3. Please note that the dopant type could be inverted (n^+^ into p-type well), as long as options are available for the guard ring. The guard ring’s purpose is to prevent undesired lateral breakdown [59,60].

The parameters set by the foundry (implantation level, atom/molecule used to dope, etc.) are serious limitations to design state-of-the-art SPADs [61]. Some groups overcame this limitation either by applying a CMOS compatible process modification (through a direct partnership with the foundry) [62] or by using a high-voltage or imaging CMOS technology [62,63]. In all cases, trade-offs must occur because the SPAD and electronics share the same technology node.

**Figure 4:**
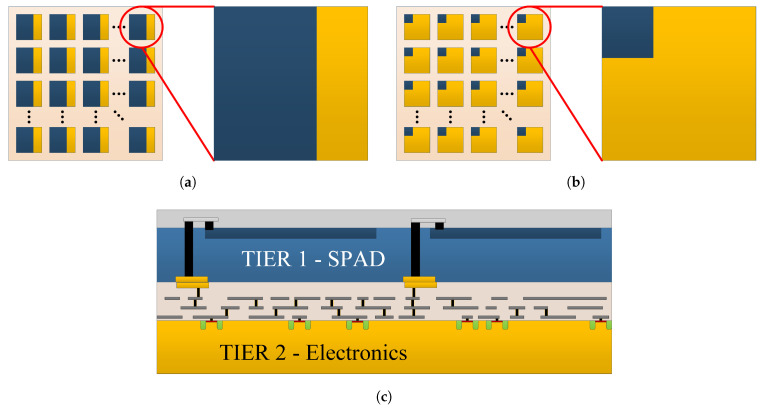
Illustration of the trade-off between the SPAD and the electronic functionality for 2D PDCs sharing the same technology node compared to a 3D PDC. In (**a**), a 2D PDC with large SPAD (blue), but limited in-pixel electronics functionalities (yellow). In (**b**), a 2D PDC with small SPAD (blue), but greater in-pixel electronic functionalities (yellow). In (**c**), a 3D PDC with large SPAD (blue) and large area for in-pixel electronic functionality (yellow).

Some 2D PDCs have large area SPADs, but limited electronic functionalities within the SPAD-QC pixel (Figure 4a) to increase the photosensitive fill-factor [57]. Others have smaller SPADs, but with more in-pixel electronic functionalities (Figure 4b) such as a counter, a time-to-digital converter (TDC), programmable hold-off time, masking of noisy SPAD, etc. [64,65]. This trade-off between photosensitive area and functionalities is inherent to the 2D architecture. In radiation instrumentation, a high fill-factor is preferred when there are very few photons or when the detection of an event’s first photons is desired [43,57]. This brings an important comment on the fill-factor: it is essential to state for a given system if the reported fill-factor includes the peripheral electronic circuits (counter, TDC, bias for monostable circuits, etc.) or if the fill-factor is provided only for the SPAD array. Each of these definitions can be a sound performance parameter depending on the application under consideration. For example, the chip level fill-factor (i.e., peripheral electronics, pads surrounding the array, space for wirebonding) must be considered for photon loss when the application requires large area detectors. This is the case for large-scale, low-background physics experiments, where large detector tiles are implemented [12,15,66].

Designing 2D PDCs in CMOS benefits from using already available technologies. It allows for rapid prototyping, avoids development cost, ensures industrial yield and reliability and provides well-established electronics design kits. Furthermore, the close integration of the SPAD-QC pair allows the reading node capacitance to be as low as possible. This reduces the charge per avalanche and thus the correlated noise (afterpulsing and crosstalk) [67]. This makes the 2D PDC an excellent option in applications where the fill-factor is less of a concern (i.e., high photon flux), such as in LiDAR sensors [68]. One of the drawbacks is related to the technology choice: with both SPAD and electronics sharing the same fabrication process, one cannot independently choose the optimal process for the SPAD (imaging CMOS, custom process, high-voltage CMOS, etc.) and the optimal process for the electronics (deep sub-micron CMOS, fast CMOS, low power CMOS, etc.).

In light of the above, there is a trend toward developing 3D heterogenous vertical integration of a SPAD array over a CMOS chip. This avoids the 2D PDC trade-offs and thus enables to reach the ultimate single-photon detector. Figure 4c is an illustration of such a 3D integrated PDC [59]. In this scheme, a high fill-factor front side illuminated SPAD array is implemented on the first tier (dedicated SPAD process). The underneath electronics (second tier) is implemented in a chosen CMOS process according to the levels of complexity and functionality needed by the application [46,48,69,70,71,72].

## 5. Review of 3D PDC

Against the odds and not without tremendous effort, the microelectronics industry managed to keep on following Moore’s law up to now [73]. Nevertheless, the strong trend known as “More than Moore” developed to add value to devices by providing functionalities that do not necessarily scale according to Moore’s Law, contributing in another way to the continuous growth of CMOS performance [74]. One avenue is 3D vertical integration, which is now democratized and used in about every field of microelectronics, from memory [75] to CMOS cameras [76] found on the most popular smartphones and tablets. In the field of radiation instrumentation, Fermi National Laboratory led in 2008/2009 a first 3D integration multi-project wafer (MPW) run where the fabrication cost was shared among participants. Two 130 nm CMOS wafers from Global Foundry were bonded face to face using Tezzaron’s Cu-Cu thermocompression process [77]. More than 10 institutions from around the globe took part in this MPW run. One of the famous successes is the vertically integrated photon imaging chip (VIPIC—Fermilab (USA), AGH UST (Poland) and Brookhaven National Laboratory (USA)), a pixelated X-ray detector [78]. This MPW run was also used to implement 3D PDC prototypes [70,79]. In sum, 3D vertical integration is a well-established technology with various processes from various vendors and can be a technology enabler for SPAD-based systems.

There are very few examples of frontside illuminated (FSI) and backside illuminated (BSI) 3D PDCs in the literature because of the complexity and the cost of the implementation. The following subsections present a review of 3D PDCs in radiation instrumentation and other fields, including the 3D PDC developed in our research group that will be explained in more detail.

### 5.1. MIT Lincoln Laboratory

The MIT Lincoln Laboratory is the pioneer of 3D PDCs. Their main interests were LiDAR and photon counting wavefront sensors. In 2002, they demonstrated an FSI 4×4 SPAD array wirebonded on top of a 16-channel front-end ASIC implemented in HP 0.5 μm CMOS process [19]. Meanwhile, the MIT Lincoln Laboratory developed their proprietary 3D integration process based on silicon-on-insulator (SOI) technology, which led them to the first 3D PDC interconnected vertically using through silicon vias (TSVs) in 2006 [71,80]. The top layer includes an array of 64×64 SPADs of 50 μm pitch. The middle tier is built in a 0.35 μm SOI CMOS technology with 3.3 V front-end electronics to control the SPAD states. The bottom tier is fabricated in 0.18 μm SOI and integrates a ring oscillator common to all pixels and a 9-bit pseudorandom counter (per pixel) for time measurement. A 3-stage delay line is also implemented in tier 2 to obtain finer timing resolution. The current challenge of this 3D PDC is the optical crosstalk mitigation, which limits the usable bias voltage [81].

### 5.2. Hamamatsu Photonics K.K.

Hamamatsu developed an FSI 3D PDC where the 32×32 SPAD array is interconnected to a 0.18 μm CMOS readout electronics using TSVs [72,82]. The octagonal SPADs are 100 μm wide with ∼50 μm wide TSV sites which provides a fill-factor of up to ∼60%. A glass handle wafer is used to hold the SPAD array wafer while it is being thinned to the required thickness. Since the handle remains after the assembly, it must be carefully chosen for the wavelength range of interest, which is the NIR in this case (LiDAR industry). The quenching circuit senses the avalanche using an inverter and features an active quenching and recharge design. A 9-bit ripple counter is also available to measure light intensity, as well as a disable bit per SPAD to shut down noisy cells. There is an adjustable hold-off time delay from 10 ns–400 ns. 32 TDCs (1 per column) are available for photon time stamping. Their most recent developments are BSI 3D PDCs for flash LiDAR, using either InGaAs SPAD array for IR wavelength [83] or their standard MPPC technology for NIR wavelength [84].

### 5.3. DESY and Semiconductor Laboratory of the Max-Planck-Society (MPG-HLL)

**Figure 5:**
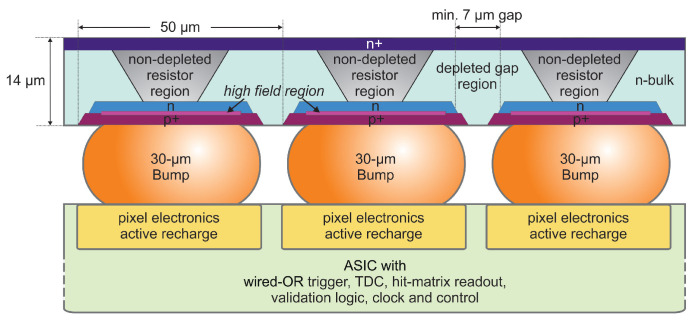
3D PDC used as a charged particle detector. (Illustration courtesy of Inge Diehl, DESY).

DESY in collaboration with Semiconductor Laboratory of the Max-Planck-Society develops a BSI 3D PDC for high frame rate multi-pixel tracking and imaging detectors for high energy physics [85,86]. Figure 5 illustrates a side-view of the 3D PDC. They also propose a similar detector for photon science applications where the SPAD layer is FSI with minimal change to the architecture presented in Figure 5 [85]. A thin 16×16 SPAD array with 50 μm pitch is bump bonded with 30 μm bumps to the readout ASIC underneath. In the initial scheme, the substrate itself acts as a quench resistor [87]. In the later version, the SPAD is actively quenched and recharged by the readout ASIC [88]. This ASIC is fabricated in IBM 130 nm CMOS. Each pixel integrates an active quenching and recharge circuit, a 2-bit ripple counter, a nanosecond range programmable hold-off time delay and a pixel-enable circuit. A fast wired-OR circuit (connected to all pixels) is used to trigger a global TDC with 77 ps bins. A combinatorial wired-OR connected to all pixels in a row triggers an adjustable threshold comparator to perform dark noise discrimination. The measured core circuit power consumption is 15 μW/pixel at 1.2 V supply and 4 μW/pixel at 3.3 V supply.

### 5.4. Istituto Nazionale di Fisica Nucleare (INFN)

**Figure 6:**
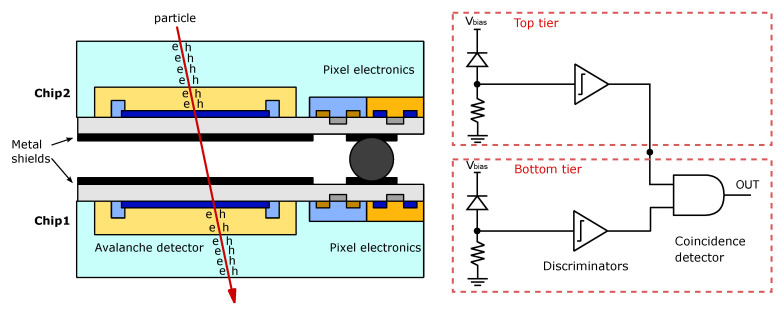
Cross-section of a 3D-PDC with SPAD on both layer for DCR mitigation using coincidence detection. (Illustration courtesy of Lucio Pancheri, University of Trento).

The INFN in collaboration with multiple universities developed a BSI 3D PDC for charged particle detection in the APiX2 experiment (note that it is not designed for visible light measurement). Compared to the other 3D PDCs presented in this section, the particularity of this detector is the use of SPADs on both the top and bottom layer of the 3D stack (Figure 6) [89,90]. Each pixel of the detector is composed of two vertically aligned SPAD to exploit the coincidence between them to discriminate between a dark count and the detection of charged particles. Their first prototype was fabricated in a CMOS 150 nm process and the two layers were bump bonded. Each pixel of the detector has a size of 50 μm× 75 μm and includes the passive quenching and the digital electronics for the coincidence measurement. The 3D PDC has 48×16 pixels and a fill-factor of 51.6%. Using the coincidence of the two-layer SPAD architecture, the detector achieved a dark count rate (DCR) as low as 24 Hz/mm^2^.

### 5.5. École Polytechnique Fédérale de Lausanne (EPFL) and TU Delft

EPFL and TU Delft presented in 2015 their first BSI 3D PDC developed for near-infrared optical tomography systems [70]. This 3D PDC is composed of two layers of CMOS 130 nm. The top layer had two arrays of 1×400 SPADs connected to an array of 1×100 processing blocks including a TDC on the bottom layer. The detector is limited to wavelengths of ~600 nm and above due to the thick silicon substrate obtained after the backside thinning process and achieved a PDE of 12% at the wavelength of interest (800 nm). In 2016, they presented a BSI SPAD-based detector using a 65 nm image sensor technology and a standard CMOS 40 nm technology [91]. Through improved backside thinning and a deep n-well SPAD structure, the SPAD achieved a PDE of 22% at 630 nm and an SPTR of 95 ps FWHM. Their most recent BSI 3D PDCs have been developed in a TSMC 45 nm/65 nm technology [92,93]. The top layer includes an 8×16 SPAD array and the bottom layer is composed of an array of QCs, a shared TDC and data processing. The SPAD array exhibits a PDE of 32% and a fill-factor of 31%. The detector is used in a LiDAR system which achieved a 430 m detection range.

### 5.6. University of Edinburgh

In 2016, the University of Edinburgh developed a BSI 3D PDC designed for time-resolved imaging using a 3D-stacked 65 nm image sensor technology and a standard CMOS 40 nm technology [94]. The top layer includes a 128×120 SPAD array with a dense 7.83 μm pitch and the bottom layer includes a QC array with a ripple counter per pixel combined with array wide data processing. The SPAD array exhibits a PDE of 27.5% at 640 nm, a fill-factor of 45% and a timing jitter between 130 ps and 220 ps. More recently, they have developed a reconfigurable BSI 3D PDC with in-pixel histogramming for flash LiDAR using a 40 nm/90 nm CMOS technology [95]. The top layer includes a 256×256 SPAD array with a dense 9.18 μm pitch. The SPAD array is coupled in a 16×1 configuration to a photon processing unit array, each of these composed of 16 QCs, 1 TDC and 1 counter. The CMOS data processing can be reconfigured to be adapted to multiple applications such as rolling shutter intensity imaging, flash LiDAR, fluorescence lifetime imaging, spectroscopy and more.

### 5.7. Université de Sherbrooke

**Figure 7:**
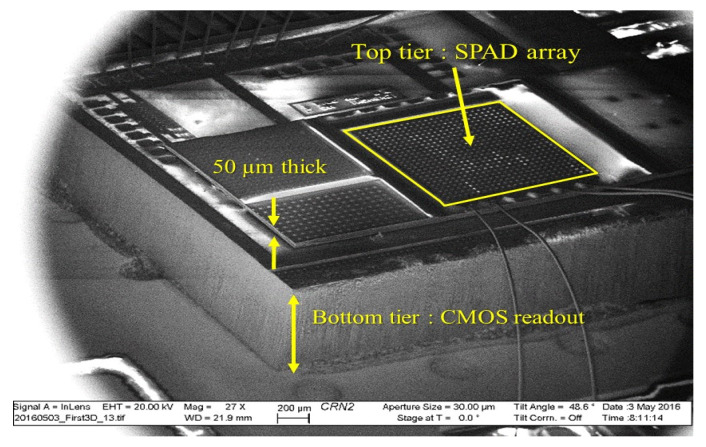
SEM image of Sherbrooke’s first 3D PDC.

Our research program focuses on FSI 3D PDCs for radiation instrumentation. Started in 2010, the work originally aimed at small animal and brain PET imaging with time-of-flight capability. Since then, the 3D PDCs are also developed for applications in low-background experiments in LXe to study neutrinoless double beta decay, namely the next Enriched Xenon Observatory (nEXO) [66,96], and in LAr to search for dark matter with the ARGO experiment. The first 3D PDC developed was presented at the IEEE NSS-MIC in 2016 based on the structure illustrated in Figure 4c. A scanning electron microscope (SEM) image of the device is shown in Figure 7.

The top layer includes a 22×22 p^+^n SPAD array with a 50 μm pitch and was implemented in Teledyne-DALSA Semiconductor Inc (TDSI) 0.8 μm CMOS process [60,97]. The implantation doses of the CMOS process were modified to improve the overall characteristics of the SPADs. After the fabrication at TDSI, the TSVs were implemented in the SPAD wafer using Aveni’s process in the U. de Sherbrooke’s facility (Interdisciplinary Institute for Technological Innovation (3IT)) clean room. Each TSV is 8 μm in diameter with a pitch of 50 μm. The distances between SPADs, the guard ring, metal interconnects and the keep-out-zone around the TSVs limit the SPAD array fill-factor between 13% and 36% depending on the SPAD architecture.

After the TSVs were implemented and interconnected to the SPAD anodes, further processing was needed before the two dies (SPAD layer and CMOS layer) could be bonded together. A handle wafer was temporarily bonded on the frontside of the SPAD array and the backside was thinned down to 50 μm to reveal the TSVs. This was followed by the insulation of the substrate and the micropillar metallization made of Ti/Cu/In [98,99]. The adhesion of the micropillar to the TSV and surrounding insulation was insufficient which resulted in few interconnected channels per 3D PDC. The working channels of these 3D devices have a peak PDE of 30% at 550 nm at 5 V of excess voltage and a DCR of ~200 cps/μ$m^{2}$. These values are comparable with equivalent 2D SPADs that have not seen any post-microfabrication processes. An equivalent conclusion was drawn from extensive testing of SPADs (with embedded electronics) that had experienced various steps of the fabrication process: TSV etching, temporary bonding, wafer thinning and bonding-like thermal cycles [98,100].

**Figure 8:**
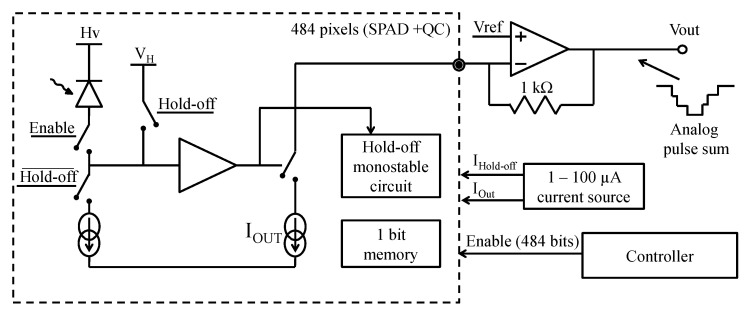
Block diagram of the front-end quenching circuit. A monostable circuit is used to control the hold-off (for afterpulsing mitigation). Also shown, the current source which is triggered when an avalanche is detected. The analog sum of each precise current source is made by a transimpedance amplifier on the printed circuit board. As shown, the current pulse width and height can be tailored.

**Figure 9:**
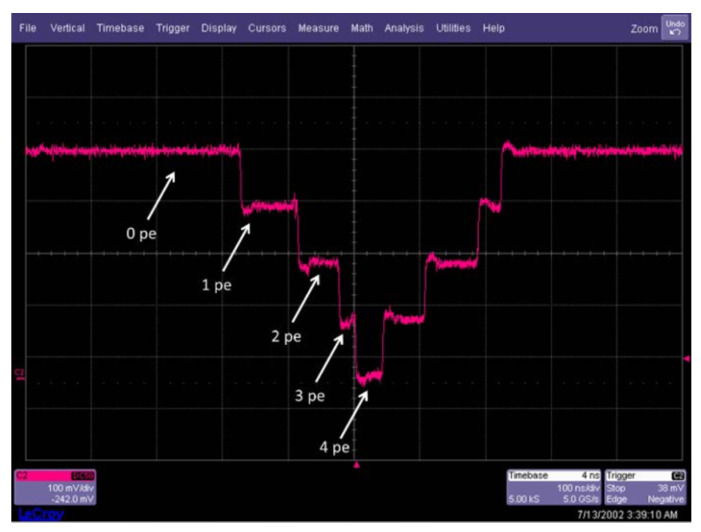
Measurement of the analog sum output of the 3D PDC. Each step represents a triggered SPAD. The current pulse width and height can be tailored.

The front-end electronics was also done in TDSI 0.8 μm CMOS and was composed of quenching circuits and an analog sum circuit, as shown in Figure 8. This analog sum produces an output signal that is similar to that of an analog SiPM. The main difference is that each triggered SPAD turns on a well-matched CMOS current source, leading to a more precise number of charges being summed at the output compared to an analog SiPM. Indeed, this masks most of the SPAD-to-SPAD parameter variations (e.g., excess voltage variations) and allows for single-photon resolution over the whole dynamic range [46]. A measured output signal is shown in Figure 9, where each downward step represents the detection of an event. The other major advantage is a lower output capacitance of the analog monitor by one order of magnitude as compared to an analog SiPM of the same area because it is only formed by the CMOS drain-to-bulk capacitance, irrespective of the SPAD capacitance. Please note that even with this analog monitor readout scheme, all the benefits of reading out each SPAD individually remain (Section 3). Finally, because this analog monitor provides good insight for debugging purposes and enables bench top experiments fairly easily, this feature should be integrated in a 3D PDC in parallel to the application dedicated readout.

In summary, although the desired system level fabrication yield was not met, a proof-of-principle was established showing that the SPADs were tolerant to the fabrication and 3D assembly process and that all key technologies were available. This endeavor provided very significant insights that led us to define design choices for 3D PDCs, as described in the next section.

## 6. Perspective of 3D PDC for Radiation Instrumentation

One of the leading arguments to move to 3D integration is to independently choose the best process for both the SPAD array layer and the CMOS readout electronics layer. This section presents key design choices that must be made to obtain an optimized 3D PDC for radiation instrumentation concerning the SPAD array, the CMOS technology, the quenching circuit, the time-to-digital converter, the digital signal processing and the system level integration.

### 6.1. SPAD Array

**Figure 10:**
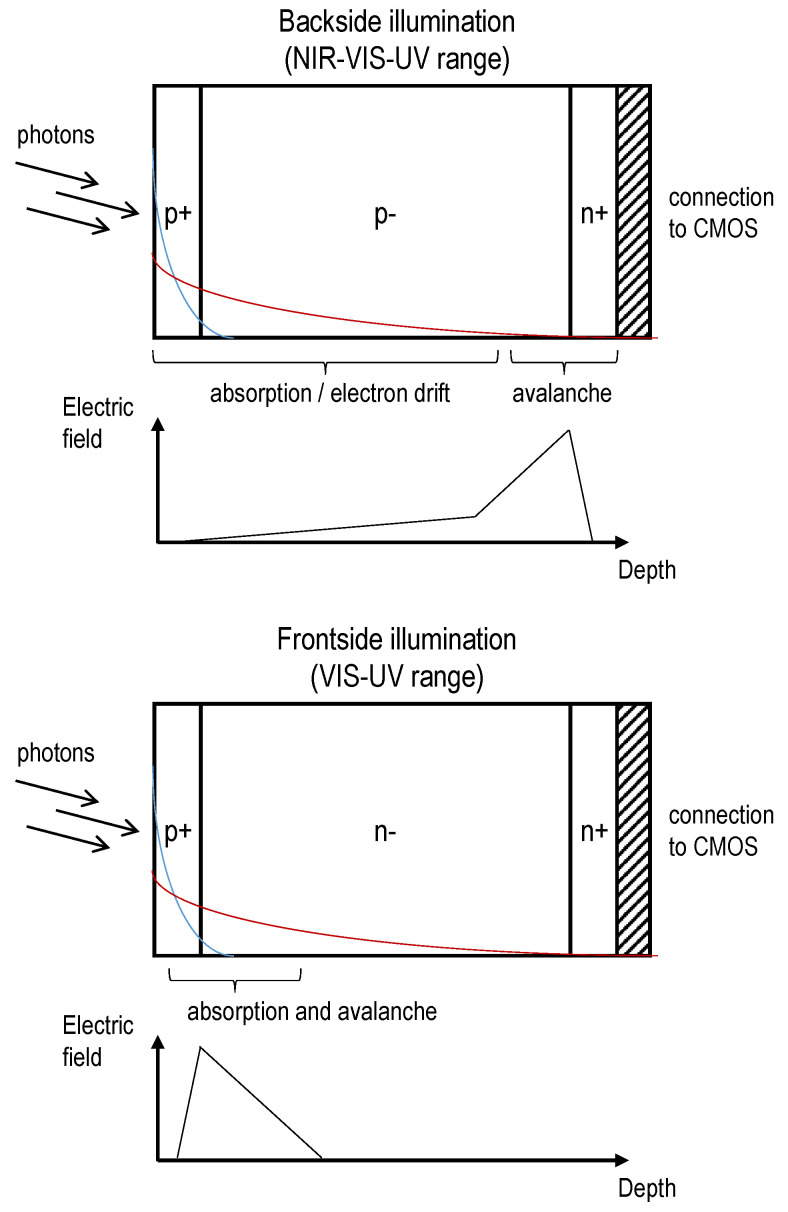
Illustration of typical backside and frontside illuminated SPADs including the different locations of the absorption/drift region, the avalanche region and a simplified electric field. The blue and red sketch lines roughly represent the typical light absorption distribution for short (UV-blue) and long (red-NIR) wavelengths, respectively.

The SPAD array is either designed in an FSI or BSI configuration. In both cases, the implementation requires freedom of design to optimize the SPAD features (junction profile, guard ring, shape and dimensions, trenches, passivation layer, etc.). This optimization can benefit from the advancements made for analog SiPMs [101]. In both configurations, the doping profiles are designed so that the photoelectrons initiate the avalanche, since in silicon the ionization coefficient is greater for electrons than holes [33]. Figure 10 illustrates typical BSI and FSI configurations. FSI refers to the SPAD junction being located on the side of the incident light similar to planar SPADs, such as in the MPD SPAD [102,103] or as in a typical 2D CMOS SPAD [57,63]. This configuration requires 3D processing to electrically connect each SPAD to the CMOS readout circuit (thinning, TSVs, backside interconnects, bonding, etc.) [72,104]. BSI refers to the junction being located on the opposite side of the incident light, near the CMOS layer. This configuration requires a bonding process to electrically connect each SPADs to the CMOS [91,94,105]. The sensor layer must be thinned down (tens of microns or lower) to detect photons from the visible range, similar to a BSI CCD [106,107].

In the BSI scheme, the absorption/drift region is distinct from the multiplication/avala nche region. The sensor cross-section is similar to a reach-through SPAD, where photocarriers must drift toward the junction before triggering an avalanche [33]. This contrasts with the FSI scheme, where those regions overlay each other. This imposes that the BSI design be fully depleted with a low-p-type or intrinsic absorption region [80,108]. In the FSI case, the structure is narrowly depleted and the profile type (p^+^n or n^+^p) must be chosen according to the wavelength of interest, depending on whether most photons are absorbed above the depletion (p^+^n ) or below the junction (n^+^p). For example, considering a junction laying at 100 nm below the surface. Detection of photons with absorption length shorter than 75 nm (λ<400 nm) favors a p^+^n junction (more than 3/4 of photons are absorbed before the junction) whereas photons with absorption length greater than 350 nm (λ>450 nm) favors an n^+^p structure (more than 3/4 of photons absorbed below the junction). The FSI’s non-depleted region induces a series resistance between the SPAD and the CMOS electronics that must be below a few kΩ [103].

**Figure 11:**
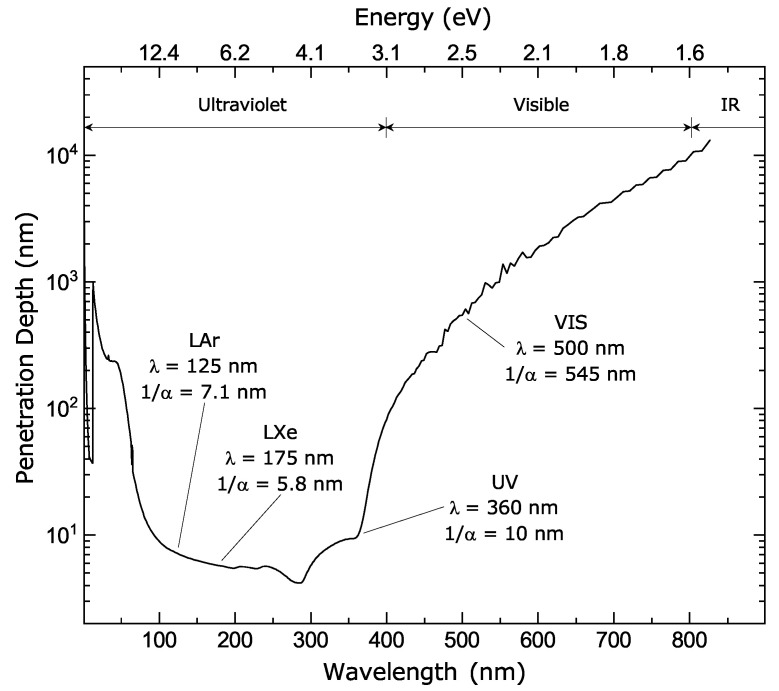
Photon penetration depth in silicon as a function of the wavelength (data taken from [111]). The minimum penetration of 4.2 nm is at a wavelength of 285 nm. The wavelengths for LXe (175 nm) and LAr (125 nm) are marked.

The main driver to use the BSI configuration is to maximize light absorption, as shown by the PDE comparison between planar and reach-through SPADs [109]. As in BSI CCDs, the BSI SPAD fill-factor tends toward unity because the illuminated side is not impaired by the presence of contact electrodes or other components such as those found in FSI devices (isolation trench, TSV, etc.). The BSI configuration is mainly used for near-infrared applications [70,95], where photons are absorbed over tens of microns. A recent work shows that some blue-enhanced/NUV architectures are also possible for the BSI configuration [110]. The FSI approach works mainly for the blue and UV range of the spectrum, since the penetration depth of these photons is below one micron (Figure 11) and therefore, inside the depleted region. Some red-enhancement architectures are also possible for the FSI configuration [102,103].

**Figure 12:**
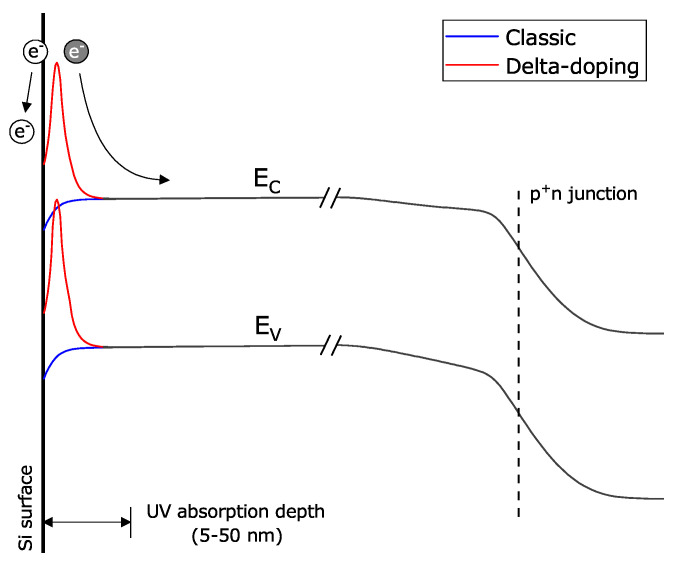
Simulation of the energy bands with and without delta-doping placed at the SPAD surface. Electrons generated past the delta-doping barrier drift toward the p^+^n junction. Only electrons absorbed too close to the surface are lost. Electrons in interface states are trapped at the surface, further reducing the SPAD dark noise.

Deep UV (<300 nm) light detection is needed in some applications, such as in low-background physics experiments in liquid argon and liquid xenon [12,15,66]. Photons below 360 nm penetrate less than 10 nm below the silicon surface (Figure 11). To maximize the PDE of deep UV photons, the energy band profile must be engineered to prevent photoelectrons from getting trapped and from recombining at the surface without reaching the multiplication region. Band profile re-engineering for UV sensitivity is a well-known process for BSI CCDs [106,112,113]. At the beginning of the 90s, the Jet Propulsion Laboratory (JPL) pioneered the delta-doping technique, a method using low temperature molecular beam epitaxy (MBE) on BSI CCDs to improve their quantum efficiency [114,115,116]. The principle is illustrated in Figure 12. For a p^+^n diode, a few atomic layers of highly boron-doped silicon are epitaxially grown on the top of the silicon to create a peak in the band profile, from 2 nm to 4 nm below the surface. This increases the probability of collecting photoelectrons that have been generated below the barrier through enhanced charge drifting. Although photoelectrons created above the barrier can still be trapped and lost, it nevertheless has the advantage that any undesired electrons thermally excited at the surface will not make it to the avalanche region, thus blocking that source of noise.

The growth of a highly doped passivation layer by MBE has also been demonstrated on BSI SPADs [117]. The deposition of a thin anti-reflection coating (ARC), optimized for near-UV light, provides a protection for the MBE layer and further increases the PDE of the detector above 70% at 405 nm. Applying this technique on an FSI 3D PDC is challenging because of the presence of surface topology (passivation layer, metal routing, etc.), as opposed to the flat backside surface in BSI detectors. To access this, one could consider performing the MBE during the sensor processing instead of the post-processing approach. Furthermore, ARCs are also needed to optimize the SPAD PDE [118,119,120]. The transmission, reflection and absorption of the passivation layer covering the SPADs’ photosensitive area depend on the wavelength and angle of incidence of the incoming photons. ARC is challenging in VUV because most materials have strong absorption, and the layers must therefore be very thin and critically controlled [121].

The main driver to use the FSI configuration is to optimize the timing resolution of the 3D PDC. The BSI absorption layer limits the SPTR of the SPAD since photoelectrons suffer from propagation time variations. This effect contributes to the timing jitter and is observed in reach-through SPADs [54,108,122]. For instance, the low-noise SLiK SPAD (i.e., a thick BSI SPAD) has a ~30 μm thick reach-through region and exhibits an SPTR of >200 ps and a PDE of 65% at 650 nm [108,123]. In contrast, a BSI SPAD made in TSMC 45 nm and thinned down to less than 3 μm has an SPTR of ~100 ps and a PDE of 25–30% at 637 nm [92]. This illustrates the trade-off between the PDE and the SPTR of the SPAD in relation to the thickness of the absorption region for a given wavelength of interest. The FSI design also suffers from this trade-off, but to a lesser extent since the absorption layer overlays the multiplication region. This allows extremely low SPTR (<20 ps FWHM), as first demonstrated with a planar SPAD in a custom process [124]. FSI SPADs made in standard CMOS technologies can also reach ultimate timing resolution (<10 ps FWHM), at the cost of high-noise and low PDE [125].

In terms of noise, in both BSI and FSI cases, the junction itself and the resulting electric field must be properly engineered to minimize DCR caused by band-to-band tunneling (BTBT) and field-enhanced Shockley-Read-Hall (SRH) generation [101]. In addition, all damage and defects in the active region must be minimized to avoid noise generation centers [103]. This is especially true for the BSI configuration, since the depleted volume is larger than in the FSI configuration [101]. DCR increases when the depleted volume is made larger [126]. The correlated noise (afterpulsing and crosstalk) is also a nuisance for both BSI and FSI configurations and is strongly dependent on the implementation (optical isolation trench, charge per avalanche, etc.). For instance, a BSI SPAD design was introduced in the mid-2000s to reduce correlated noise while maintaining a large fill-factor [127]. This design keeps the absorption layer unchanged and reduces the junction’s diameter to minimize the charge per avalanche and hence the afterpulsing and crosstalk noise. Electric field engineering is needed to funnel the photocarriers into the point-like multiplication region.

In terms of light detection, both BSI and FSI are suitable for radiation detection applications (visible, UV and VUV range). Because of the better achievable fill-factor, the BSI design has a slight edge over the FSI for rare signal/low-background applications such as experiments in noble liquids [12,66]. Because of the better achievable SPTR, the FSI design is relevant for precise timing experiments and ToF applications such as ToF computed tomography [128] and ToF-PET [129]. Achieving key characteristics (high PDE, optimal SPTR and low noise) brings design trade-offs and the different applications in radiation instrumentation require the pursuit of both BSI and FSI 3D PDC implementations.

### 6.2. 3D Vertical Integration

One of the leading arguments to rely on 3D vertical integration is the possibility to choose the best process to design and fabricate the SPAD array and to independently choose the most appropriate CMOS process for the readout electronics. Optimal technologies will bring optimal performances of both SPADs (PDE, noise, SPTR) and CMOS electronics. However, more than this obvious argument, the 3D integration allows for higher fill-factor, higher degree of integration into systems (e.g., large-scale photodetectors) and for a less capacitive and more uniform connection to the SPAD to reach ultimate performance in timing resolution and/or power dissipation.

Although the SPAD technology is selected as described above, the CMOS process is chosen based on the complexity of the desired readout electronics per SPAD, the compatibility with 3D integration, the availability of MPW to prototype the CMOS prior to purchasing wafers for 3D assembly and the cost. Advanced 3D integration processes usually require the handling of full wafers (as opposed to die-to-die assembly techniques). Purchasing complete CMOS wafers (as opposed to sharing in multi-project runs) is expensive and limits R&D to only the big players of the industry, large institutions such as national laboratories or to large-scale physics experiments. There are ample methods to perform 3D integration (molecular bonding, microbump bonding, direct bond interconnect, etc.) [130,131]. To give a good measure of the level of integration that they allow, recent progress in microbump bonding can accommodate 16 μm to 20 μm bumps with 35 μm spacing, which is reasonable for a SPAD pitch varying between 50 μm and 80 μm.

A game changer for the development of 3D PDCs would be to identify a 3D assembly process that could be done at chip level (i.e., not requiring complete wafers) to cut cost. In a similar line of thought, one could use a design of SPAD array (mainly a generic geometry with ARC and PDE optimized for a specific application) and mate it with any CMOS readout electronics with the proper pitch and application specific functionalities. Such an approach would allow researchers interested in 3D PDCs to cooperate, identify a CMOS technology and a 3D bonding process, and develop the SPAD array with an industrial collaborator. This could lead to cost sharing a multi-project 3D PDC process run in which each team would have their custom readout circuits for specific applications.

### 6.3. CMOS Process Choice

The selection of the CMOS process should take into account the SPAD characteristics, such as the bias excess voltage the front-end electronics will need to quench, the size of the SPAD wafers to match with the CMOS wafers for 3D integration, the SPAD size constraining the size of front-end electronics for each SPAD, the complexity of the desired digital signal processing embedded in the ASIC, the cost and the availability of MPW runs. For instance, in the specific case of ToF-PET imaging, a TDC with sub-10 ps FWHM is desirable and points toward more advanced CMOS processes [48,129,132,133]. In the case of noble liquid detectors, very large area must be covered with maximum fill-factor and minimal power consumption to prevent boiling and convection of the noble liquid [12,66]. In Section 6.6, these two examples will be presented with detailed arguments for the selection of the CMOS process. When the system level fill-factor is critical, the PDCs should be 4-side tileable. In such systems, TSVs should replace wirebonds to connect all input and output signals from the backside of the CMOS tier to the printed circuit board or silicon interposer. The TSV technology is commonly used in high-end microelectronic devices, but is often vendor specific thus limiting the selection of the CMOS technology or limited to wafer level processing.

### 6.4. Quenching Circuit

The QC is composed of three main parts: the quenching and recharge branch, the sensing and discriminating circuit and finally the monostable circuits to set the hold-off time and recharge time for afterpulsing mitigation. The literature on QC architecture (in 2D) is quite extensive and readers are referred to these two articles as a starting point [22,32].

For the quenching and recharge branch, multiple architectures are present in the literature: traditional passive, active or mixed (passive quenching–active recharge, variable load quenching, etc.) [22,32,67,91,125,134]. The main challenges for the quenching and recharge branch are to allow for a maximum SPAD excess voltage for the PDE while minimize the capacitance on the node for the timing and noise. The quenching circuit will need to swing the SPAD electrode from the excess bias voltage to below breakdown. The voltage breakdown of the MOSFET’s drain junction to the CMOS substrate limits the voltage excursion and care must be taken when choosing the technology. One option is to use a CMOS process with HV devices, allowing for a greater voltage swing, but at the cost of larger area CMOS HV devices, limiting the available real estate for the other transistors of the quenching circuit. In recent years, new architectures have been proposed to allow a greater excess voltage through different implementation of cascode transistors [48,91,135,136]. For example, one architecture allows for an excess voltage as high as 3 times the maximum voltage of the CMOS technology [136]. Please note that the impact of these cascode-based architectures on the timing jitter of the SPAD-QC pair has not yet been demonstrated.

To reduce the timing jitter, the capacitance at the node of the detector should be minimized to increase the slope of the signal. A mixed architecture such as passive quenching–active recharge or a variable load quenching allows for minimization of the capacitance directly connected to the SPAD, as compared to a full active quenching circuit, by reducing the number of required transistors at the reading node [125]. On the other hand, to reduce correlated noise and power consumption, both minimizing the capacitance and stopping the avalanche before the complete discharge of the SPAD limits the number of carriers involved [67]. To this end, the sensing and discrimination node must sense the avalanche to provide a positive feedback to the quenching branch to quench the avalanche as soon as possible. As the afterpulsing and optical crosstalk are functions of the number of charges involved, the QC should swiftly detect the avalanche and stop it, an advantage of active quenching circuits over passive implementations.

For the sensing and discriminating circuit, the QC acts like a classic leading-edge discriminator that can be implemented with a simple inverter. An inverter has the advantages of having low power consumption and taking a low area. In a 3D architecture, more real estate is available and a comparator with an adjustable threshold can be implemented to obtain a better SPTR [137]. One of the contributions to the timing jitter is given by the ratio of the signal noise over the slope of the signal at the discrimination point [125]. Being able to set the threshold at the optimal value is the advantage of a comparator-based architecture over the inverter. Considering this, in a 3D PDC optimized for large area detectors where the power consumption is a critical requirement, an inverter is preferred. On the other hand, for an SPTR optimized 3D PDC for PET, a comparator should be implemented [48].

Multiple comparator-based QCs have been developed to optimize the timing jitter. In [137], they report that excellent SPTR (35 ps FWHM) can be obtained if the avalanche is detected at low levels while the multiplication process is still confined to the photoelectric interaction point. Following that, multiple SPAD-QC pairs with a comparator were developed to reach an SPTR of 27 ps FWHM [32], 20 ps FWHM [138,139] and finally 7.8 ps FWHM [125]. The adjustable threshold is also handy for characterization purposes: scanning the threshold over the input dynamic range provides information on SPAD voltage breakdown variations and the risetime of the SPAD signal [125,140]. For example, studies show that the excess voltage of a single CMOS 65 nm SPAD can vary up to 30 mV FWHM [125] and up to 60 mV FWHM for a CMOS 180 nm SPAD [141]. This provides important information: the QC must have low propagation delay variation as a function of the SPAD signal amplitude variations as it gets convolved with its timing jitter [125]. Additionally, in an array configuration, the QCs must have uniform routing propagation delay because any variation gets convolved with the system timing jitter. If the SPAD address is recorded, the digital signal processing unit of the ASIC can correct for the routing propagation delay variation and align the mean of the timing spectrum of each channel using calibration data stored in an on-chip lookup table (as discussed in Section 6.6.1 [48,69].

Each QC should include two monostable circuits: one that implements the hold-off delay used to minimize afterpulsing, and a second that gates the QC output until the SPAD is fully recharged to prevent spurious triggering and ensure a more uniform SPAD excess voltage while operating. Finally, the possibility to enable or disable each SPAD of a PDC opens doors to new characterization methods not possible with analog SiPM. For instance, one will be able to study the impact of optical crosstalk as a function of the relative position with respect to the emitting SPAD or characterize a single SPAD within an array [55].

### 6.5. Time-to-Digital Converter

Many detectors in radiation instrumentation require the time of interaction and to obtain it, multiple circuits can be implemented. For a rough estimate, a counter on a clock signal can provide the basic information needed. For very high timing precision, a TDC allows for precise measurement between a start and a stop signal. Key parameters of a TDC are the timing jitter, the least significant bit (LSB), the area, the power consumption and the conversion time.

In the field of radiation instrumentation, there is a trend to reach sub-10 ps FWHM timing jitter for applications such as PET imaging using prompt photons [129], ToF computed tomography [128] and time-resolved calorimetry. It is quite a challenge to achieve a TDC with such an LSB and jitter while having a small area and a low power consumption, two parameters required in large systems such as a PET scanner.

The TDC LSB represents the smallest time step the TDC can measure and has a direct impact on the timing jitter through the quantization error (LSB/$\sqrt{12}$ RMS). For ToF-PET, the sub-10 ps FWHM timing jitter sets the LSB to about 5 ps, limiting the types of TDC architecture that can be implemented. The conversion time of the TDC must be as short as possible to minimize its dead time, the time interval during which it cannot timestamp another event. Please note that this can be mitigated in certain applications such as ToF-PET. For example, during the time a TDC is converting its timing information to a digital format, the circuit can still count the number of times the SPAD-QC is triggered, hence minimizing the impact of the device dead time and providing a way to count all photons (e.g., for energy measurements) [48].

Limited by real estate, most 2D architectures rely on queuing theory to implement time-to-digital conversion within groups of SPADs [142,143]. Although this was shown to be functional, the ultimate coincidence timing resolution (CTR) can only be met by using one TDC per SPAD or per small group of SPAD-QC pairs to maximize the probability to timestamp the firsts prompt photons [144]. In theory, one TDC per SPAD-QC seems the best choice since it allows for correction of the timing skew of each pixel of a SPAD-QC-TDC. However, it comes with many challenges such as limited area (<50×50 μ$m^{2}$) and power consumption per TDC (< 100 μW) [133,145].

If one TDC per SPAD-QC pair is required, it must be relatively small to fit alongside the QC and other circuits under the SPAD real estate with the same footprint. Combining this requirement with sub-100 μW, 10 ps FWHM jitter and 5 ps LSB limits the TDC architectures that can be used. One of the prevalent architectures to reach both a small area and a small LSB is the single stage Vernier architecture [146,147]; however, work is still required to reach the 4 combined key parameters. For example, Vernier ring oscillator TDCs can achieve low area (<25×50 μ$m^{2}$) and low power consumption (< 22 μW) with a 5.1 ps LSB, providing a timing jitter of about 13 ps FWHM [133,145]. The same architecture is implemented in an array of 256 TDCs per 1 m$m^{2}$ to achieve a one-to-one coupling and the resulting timing jitter for the whole array is about 40 ps FWHM (18 ps RMS). This timing jitter degradation is mainly due to non-uniformities between TDC LSBs and common mode noise injection from the number of TDCs running on-chip [48]. To minimize the common mode noise, one could implement one TDC per small group of SPAD-QC pairs and still apply corrections for the timing skew of each SPAD by adding an auxiliary circuit such as an arbiter to identify the SPAD that triggered [48]. The challenge is to design an arbiter with sub-10 ps timing precision with multiple inputs (i.e., the number of SPADs linked to a single TDC). That being said, the brute force approach of providing one TDC per SPAD is most likely excessive for many applications and a careful study must be performed to identify the right ratio of SPADs per TDC to meet the specifications. For example, if timing is not critical (>250 ps) for a specific application (e.g., nEXO), then there are other TDC architectures or other TDC implementation schemes that can be considered. This does not discard the other advantages of the 3D architecture: optimizing the number of SPADs per TDC frees real estate for TDC improvements and advanced signal processing.

With respect to timing resolution, 3D integration allows for a SPAD of ideal geometry (particularly a uniform current collection) to be integrated at a short and uniform distance from a low jitter quenching circuit (e.g., comparator-based) with a stabilized low jitter TDC and further allows for signal processing to use address-based calibration data to compensate timestamp non-uniformity.

### 6.6. Digital Signal Processing

As pixels are getting smaller and systems (scanners or physics experiments) keep growing in size or density, the amount of data to gather and process is also becoming an issue. With 3D PDCs, various signal processing can be implemented at the sensor level to filter, select, condition and compress the data to limit the required bandwidth, and hence save on the system power consumption.

In 2D, the digital signal processing will require some area on the periphery of the SPAD-QC array therefore losing system level photosensitive fill-factor. This is more easily mitigated in a 3D PDC because signal processing is done by the CMOS tier under the SPAD array. For instance, if the QC and the TDC combined area is smaller than the SPAD area, digital signal processing and data transmission circuits can be distributed in each pixel of the ASIC, not impacting on the system level fill-factor.

The embedded digital signal processing is tailored to an application and can be very simple such as photon counts within a time window, or highly sophisticated such as multi-timestamp estimator with inline correction. To exemplify this, two application cases will be explored: (1) a preclinical/brain time-of-flight PET (ToF-PET) scanner with tight requirements on SPTR, and (2) a 3D PDC designed for a large-scale integration (multiple meter square) to operate at cryogenic LAr and LXe temperature.

#### 6.6.1. Time-of-Flight PET Scanner

**Figure 13:**
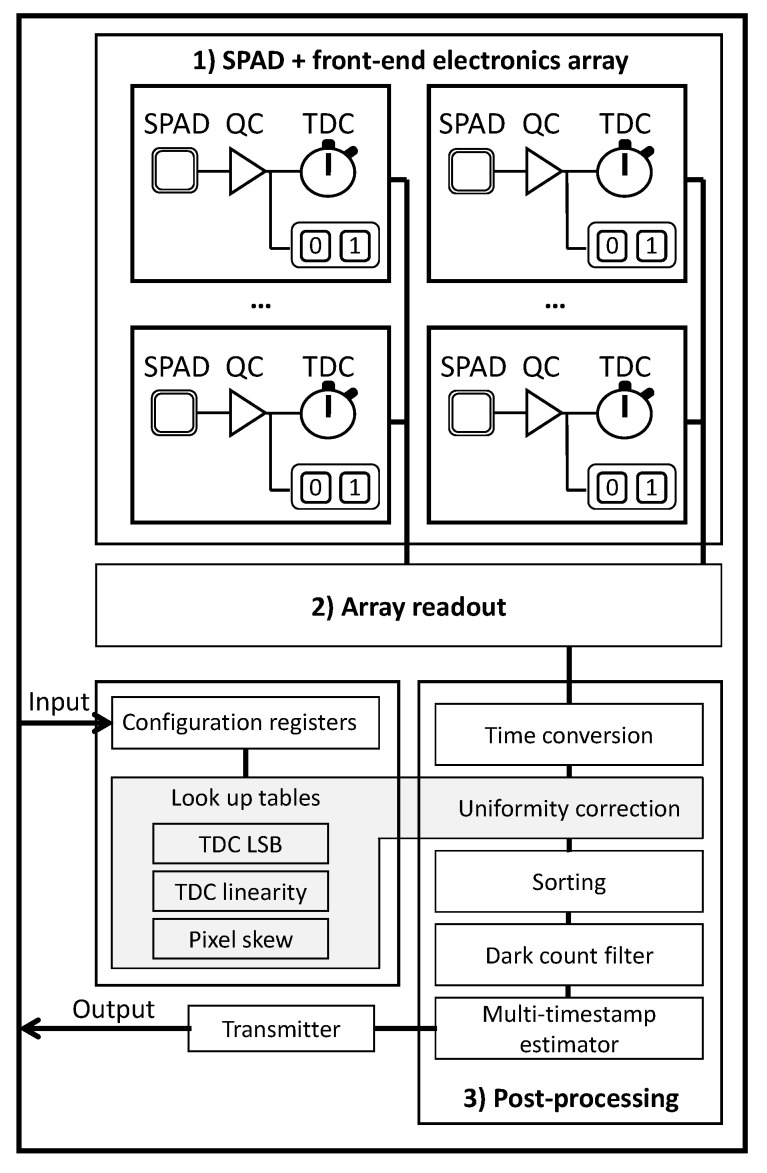
Block diagram of a 3D PDC dedicated to PET. (1) The SPAD, QC, TDC and counter array; (2) An array readout to send the information to the digital signal processing; (3) The digital signal processing with TDC uniformity correction, timestamp sorting and filtering and a time estimator based on multiple timestamps.

Figure 13 presents the block diagram of the digital signal processing required for PET imaging with the goal of eventually reaching sub-10 ps FWHM array wide [48,69]. In ToF-PET, a burst of prompt photons followed by scintillation photons with a sharp rise time (sub-ns) and a long decay (tens of ns, depending on the crystal used) should be measured. The role of the readout is to timestamp the firsts prompt photons and to count all following photons during a given time interval. The timestamps are used to identify coincidence between PET events and the photon count gives the energy of the measured gamma to discriminate Compton events.

To prevent acquiring dark noise and therefore reducing the data bandwidth, a dark noise discrimination filter must be implemented; the acquisition is started only when a certain number of columns (programmable threshold) in the array have at least one trigger within 6 clock cycles [48]. If the threshold is not met, all TDCs are reset to be ready for another event. Other implementations of dark count filters can be found in the literature [57,79]. Filtering out dark noise is a major advantage of PDCs that reduces the output bandwidth and power consumption, knowing that a significant amount of power is lost and dissipated in the data transfer between electronic circuits.

When an event is detected, the whole array is then read out by the digital signal processing module, where the address, timestamp and number of counts are stored. To correct for the time skew between pixels and the TDC LSB non-uniformity throughout the array, a calibration is performed when initializing the ASIC and the correction values are stored on-chip in lookup tables [48,69]. These would be difficult to embed in a 2D implementation since lookup tables require large on-chip area. Once the timestamps are corrected and stored, a sorting engine puts the timestamps in chronological order. At this point, a second dark count filter goes through the ordered timestamps and removes the timestamps before the event that are most likely due to dark counts and not photons. The dark count filter is used to increase the multi-timestamp time estimator precision [69]. To complete the signal processing chain, a best linear unbiased estimator (BLUE) [69,148] is used on the *n* firsts photons to extract the timestamp of the 511 keV event. To estimate the improvement of this post-processing scheme, hardware-in-the-loop simulations were performed using a fabricated ASIC [48] and simulated PET event (LYSO scintillator and SPAD) [149] which showed a coincidence timing resolution (CTR) improvement from 160 ps FWHM to 126 ps FWHM and a bandwidth reduction from 34.7 Mbit/s to 0.5 Mbit/s [69].

As for the energy of the event, it was shown that the same timestamps can be used to discriminate between a real event and a Compton event [150]. The number of emitted photons by the scintillator increases with the energy deposited. Assuming that the rise time and decay time of the scintillator are independent of energy, the amplitude of the signal increases with the number of detected photons. Hence, the time interval between two detected photons is smaller for higher energy events, and oppositely the time interval will increase if the PET event has less energy. To discriminate in energy, the time interval $t_{k}$ between the first detected photon and the photon of rank *k* is used. This is compared to a programmable threshold $T_{k}$. If the time interval $t_{k}$ is greater than $T_{k}$, then the event is discriminated in energy as a Compton event and rejected, but if the time interval $t_{k}$ is smaller than $T_{k}$, then the event in conserved. All the signal processing described above is performed on-chip.

#### 6.6.2. Liquid Argon and Liquid Xenon Experiments

Some neutrino [96,151] and dark matter [13,152] experiments rely on the measurement of faint scintillation photons in large volumes of noble liquids. They thus require a large area photodetector system operated at cryogenic temperatures capable of counting single photons. Indeed, 10 to 10,000 photons are to be measured per event and collected over many square meters of detectors [66]. The scintillation light wavelengths of LXe and LAr are in the VUV range (175 nm and 125 nm respectively), but can be converted to visible light with the use of wavelength shifters (mainly in LAr experiments). Another requirement is the reduction of contaminants (mainly organic) that affect the light yield and the secondary electron lifetime in experiments with time projection chamber (TPC) configurations (e.g., nEXO). These experiments are also labelled as “low-background” because great care is taken to select all construction materials to limit spurious decays in a specific range of energies. Having the detector directly in the scintillation medium makes this constraint even more stringent on radio purity. Fortunately, silicon has been shown to comply with this requirement.

LAr and LXe experiments are real technological and economic drivers for 3D PDCs due to their large scale (nEXO, DUNE, ARGO) [10,11,12,13,14,15]. As many functionalities are similar for LXe and LAr experiments, one could design a 3D PDC based on the same CMOS process, 3D integration approach and SPAD array that would support the various experiments worldwide. Still, this approach permits having tailored digital signal processing. However, in all cases, as the 3D PDC would be installed within the noble liquid, one of the main design criteria is low power consumption, one reason being to avoid convection in the time projection chamber.

**Figure 14:**
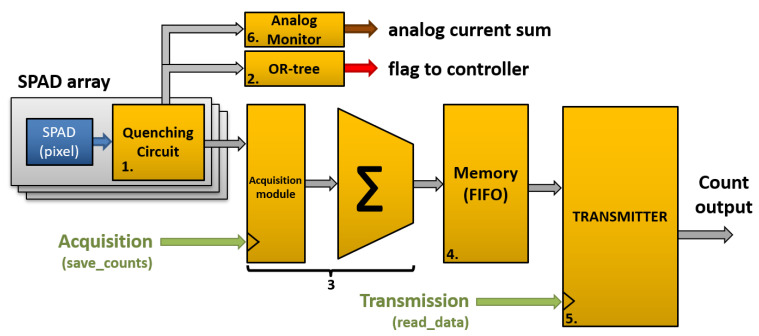
Block diagram of a 3D PDC dedicated to LAr and LXe. (1) The SPAD and QC; (2) A flag is set as soon as a photon is detected on any SPAD; (3) The acquisition module saves the actual count of triggered SPADs; (4) The FIFO saves the number of counts as a function of time; (5) The transmitter sends the counter’s value to the acquisition system; (6) The analog current sum is proportional to the number of detected photons, as shown in Figure 9.

For example, one can design a readout ASIC prototype that operates in two modes dedicated respectively to the nEXO experiment [96] and to the ARGO experiment. In Figure 14, a block diagram is shown of the ASIC fabricated in TSMC 180 nm BCD (Bipolar-CMOS-DMOS) process. The ASIC readout implements an inverter-based quenching circuit per SPAD. Programmable hold-off and recharge delays are provided for the whole array. When a SPAD is triggered, the information can be read by means of 3 output types: (1) a fast, low jitter, global OR-tree flag signal that provides the means to timestamp the triggering event with an external TDC; (2) an analog monitor (see Figure 9); and (3) a digital data processing unit detailed below for the two applications.

The dedicated nEXO (LXe) mode focuses on power consumption reduction by asynchronous operation: when there are no photons, the power consumption is below 65 μW (static power consumption and leakage) for a 5 × 5 m$m^{2}$ array. Each 3D PDC continuously monitors all SPADs and sends a digital flag to an external tile (group of devices) controller when a SPAD is triggered. The controller can count the number of triggered SPADs in a programmable time window (typically 200 ns) using the 3D PDC flag signal. When this number exceeds a programmed threshold, a clock will be sent temporarily to execute the digital sum and to enable this data exchange between each 3D PDC and the tile controller, keeping digital switching to a minimum. Please note that by knowing the limited number of photons emitted and the large volume of the experiment, it is likely that the detected photons will be distributed among many 3D PDCs and a decision to read out all channels would come from the external DAQ system. If an event occurs close to the photodetector tiles, then many 3D PDCs within a given area will count many photons, justifying the flexibility of this functionality.

The LAr mode is designed to perform pulse shape discrimination (PSD). Pulse shape discrimination in LAr makes use of the difference in scintillation decay time between nuclear recoil events and electronic recoil background events. Event discrimination is based on the fraction of light detected in the first tens of nanoseconds of an event with respect to the whole event duration (∼μs). It allows for rejection of otherwise dominant backgrounds from beta and gamma radiation at the $10^{-7}$ level [152]. In LAr mode, the SPAD-QC cells still asynchronously perform the avalanche monitoring, quenching, hold-off and recharge phases. The difference with the nEXO mode is that a clock sets a fast frame rate (10 ns minimum). The digital adder result is pushed into a 128-bit deep FIFO that stores the number of counts in bins of configurable width (multiples of the 10 ns clock period). When an event is identified by the tile controller and signaled back to the 3D PDCs, the device stores a set number of short bins then switches to storing longer time bins for the rest of the event duration. All these are configurable parameters. This gives access to the relevant signal waveform to perform PSD. In addition, the low jitter OR-tree signal can be used with a TDC to timestamp the event. A virtual fiducial volume can thus be created using time-of-flight, provided that the peak-to-peak timing jitter lies between 200 ps and 1 ns. The TDC could be implemented in the 3D PDC or in the tile controller.

### 6.7. 4-Side Tileable

Having in mind the aim to maximize the system photosensitive fill-factor, future 3D PDCs should imperatively be 4-side tileable so that they can be assembled with a minimum loss of system fill-factor. This requires that all input and output signals (IO) of the 3D PDC be connected from the backside of the device making use of TSVs. As an example, due to having only one tier of CMOS electronics, imaging integrated circuits have IO TSVs integrated from the backside by post-processing of the CMOS wafers to connect to a redistribution layer and bonding pads. Illustrations of the concept are shown in [153]. In recent years, multiple companies and research groups developing analog SiPMs have added TSVs for IO to maximize the system integration fill-factor for large-scale experiments [154,155,156].

Please note that the main contribution of TSVs is to free the space around the device required for wirebonds. However, IO TSVs and their protection still take a significant amount of space in the circuit layout. When the TSVs are used in analog SiPMs, they take photosensitive area. In a 3D PDC, the IO TSVs are integrated in a CMOS readout chip and can be confined within the same area as the SPAD array. To help fit all the required electronics under a SPAD (the QC and other functionalities), finer deep-submicron technologies can be used, but the cost of these technologies becomes rapidly prohibitive. To solve such issues, Fermilab used two tiers of 130 nm CMOS: one for analog circuits bonded to the sensor, and one for digital circuits with IOs and IO protections [157,158]. The final integrated circuit is a 3-tier system. With this, all IOs and peripheral circuits are distributed on the digital bottom tier making 4-side tileable devices possible.

### 6.8. Tiles for Large-Scale Detector in Cryogenic Operation

To build large-scale detectors, the 3D PDCs need to be assembled into tiles, grouped units of 10 c$m^{2}$ to 100 c$m^{2}$ [14,66]. The tiles must efficiently pave the detector to maximize the photosensitive area, provide on-site signal conditioning and send data to the external data acquisition system. Experiments such as nEXO can only tolerate a low radioactive background and a low number of impurities which prohibits the usage of PCBs made of FR4 or any common organic laminated materials [66]. To improve the reliability and minimize the induced stress at cryogenic temperature, the coefficient of thermal expansion (CTE) of the tile should be closely matched to the 3D PDC’s CTE [159,160]. Interposers are good candidates to replace the functionalities of a PCB in low radioactive cryogenic particle physics experiments. Most of the commercially available technologies limit the tile area below 10 c$m^{2}$. With the large interest in 2.5D/3D electronics, interposers are a hot topic in the microelectronics industry. Indeed, they offer a level of integration between a PCB and an integrated circuit [161]. Glass and quartz interposers have great interest by the research community because of the high substrate isolation that increases RF capabilities compared to lossy silicon substrates [162,163]. Even if glass and quartz substrates are good candidates for RF transmission, large area commercial interposer applications are limited with those materials [164]. Fabrication over silicon substrates is a well-understood process by the microelectronics industry which allows the fabrication of small silicon interposers with multiple redistribution layers (RDLs). As for cryogenic operations, the usage of silicon as the tile core allows for matching of the CTE with the 3D PDCs.

To minimize complexity, yield and development risks, passive interposers are a baseline technology. However, it is most likely that future requirements (5–10 years) would benefit from built in active circuits. For instance, these active interposers could embed voltage regulators, digital-to-analog converters, line drivers, signal equalizers, decoupling capacitors or even phase-locked loops. Active interposers can support distributed electronics without reducing the circuit density. The development of a large area multi-RDL silicon interposer, be it passive or active, could very well provide the system integration necessary to create the required 3D PDC tile for particle physics experiments.

### 6.9. Implementation Challenges

Implementing 3D PDCs comes with its load of challenges. As the SPAD and the CMOS are not implemented in the same fabrication processes, one needs to prototype both independently. The CMOS technology must be chosen early in the project according to the excess voltage to be quenched and the digital signal processing circuit requirements, but also according to 3D assembly constraints. Then, the readout circuit can be fabricated by itself to validate all functionalities and performances without the SPAD array bonded to it. To have realistic input test signals, SPADs can be integrated directly into the readout CMOS technology. This is the opportunity to develop all the ancillary electronics needed to communicate, configure and control the readout electronics prior to having a 3D device.

**Figure 15:**
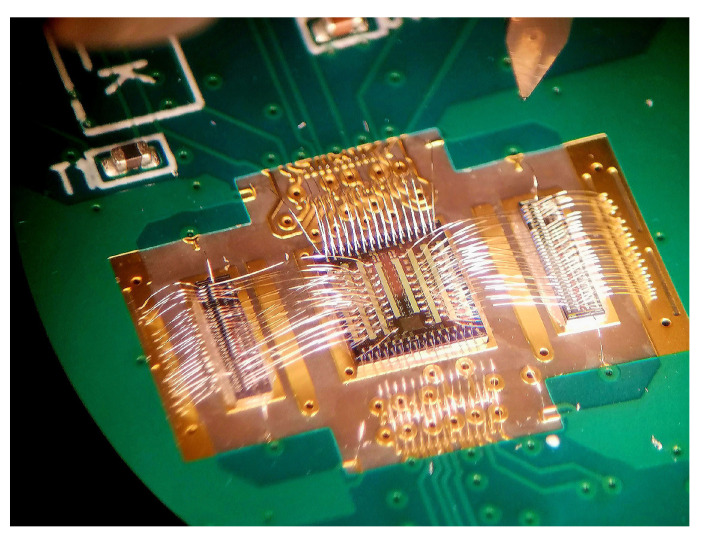
In the middle, the readout ASIC with an array of QC. On both sides, strips of various flavors of SPAD under study during fabrication process optimization.

The challenge for the development of the SPAD array layer of a 3D PDC made in a custom process is the unavailability of transistors in the layer, and hence no quenching circuit to test them. There is no straightforward way to infer all the SPAD characteristics (i.e., in Geiger mode) from DC measurements, in particular timing resolution. To overcome this issue, we have designed a dedicated integrated circuit (dubbed “ChipProbe” [141]) with arrays of quenching circuits made to readout SPADs that are connected either in a flip-chip configuration or by wirebonds as shown in Figure 15. The quenching circuits have been specially designed to overcome the added capacitance that the SPAD-to-ChipProbe interconnection brings. They allow for the study of SPADs at various excess voltages and threshold levels to explore all relevant parameters for PDE, noise and timing performance. All this accounts for a relatively long design cycle before having a 3D PDC (or any other imaging sensors in 3D).

By basing the device development on established process flows, recipes, capabilities and tools from the partner foundry, key risks must be identified and mitigation for them must be provided. For example, short fabrication loops are used to assess the existence of issues with the process and to provide solutions if needed. It is imperative to have strong ties with a foundry as the process flow will be based mainly on the foundry’s recipes, capabilities and tools available. Also, compared to the CMOS which can be prototyped at relatively low cost through MPW runs, developing a SPAD array in a commercial foundry requires significant funding. Another variable is the time required. Even with decent funding, it is not trivial to be a high priority project when compared to other commercial products being fabricated in the foundry.

## 7. Conclusions and Outlook

The goal of this paper was to shed some light on the potential technology successor of analog SiPMs in radiation instrumentation. 3D PDCs can be seen as the natural evolution of SiPM technology. A comprehensive literature review of SPAD-based systems was presented, with a bias toward radiation instrumentation. A PDC provides a better single-photon resolution and SPTR by reading each SPAD individually in addition to the hold-off and enabling/disabling functionality to minimize the noise. Furthermore, the digital approach allows for embedded dedicated digital signal processing tailored for the application. The PDC reaches its full potential through 3D integration to maximize its fill-factor, but also by allowing optimized technologies for both the SPAD and the electronics.

The power consumption comparison between the analog front-end of the SiPM and the digital circuit of the PDC is not trivial. These two approaches lead to different output format, area coverage and key characteristics. To obtain a fair comparison, the two architectures need to be compared at the system level while considering their overall performance (for example pixel size, system fill-factor, timing, noise, etc.).

A 3D PDC can be either frontside or backside illuminated. As presented, both architectures are relevant for different purposes/applications. A frontside illuminated detector has more potential regarding the timing characteristics, but the integration process is more difficult. On the other hand, a backside illuminated detector has a better PDE at the wavelength of interest due to a higher fill-factor and a flat surface which allows for an easier surface treatment. This higher PDE comes at the price of a degraded timing jitter.

An extensive discussion on the key parameters of the various blocks of the embedded digital signal processing found in a PDC was presented. For high timing applications (e.g., ToF-PET), each SPAD should be coupled to a comparator-based QC and a high-precision TDC. Furthermore, the embedded digital signal processing must correct the non-uniformities of each SPAD, QC and TDC to reach a high timing precision. In the case of noble liquid detectors, each SPAD should be read by a low power QC and the digital signal processing should operate asynchronously to minimize power consumption.

Developing a SPAD array and 3D assembly in a commercial foundry requires significant funding and time, but yields the production capabilities for large volumes of devices. The envisioned development of large-scale physics experiments such as nEXO and ARGO calls for a significant volume of devices to be produced and assembled. We believe there is room for concerted efforts to develop the enabling technologies for these experiments. As seen with other technologies, these experiments could be a driving force that would help to democratize 3D PDC technology for other applications that do not offer the same volume of production. For example, a 3D PDC MPW can be used as a starting point to democratize this technology and provide access to new design ideas. In this concerted effort, the SPAD array wafer, the 3D bonding technology as well as the same CMOS wafer would be shared between groups taking part in the collaboration and each group could integrate the specific functionalities for their needs in the CMOS circuit. Another game changer for the development of 3D PDCs would be to identify a 3D assembly process that could be done at chip level, not requiring complete wafers. Our wish is to design SPAD arrays optimized for radiation instrumentation and mate them with any CMOS readout electronics with the proper pitch and application specific functionalities. This could lead to cost sharing in a multi-project 3D PDC process run. The next step for the 3D PDC is a tileable integration in a photodetection module to have a workable device to be integrated in a real system.
